# Prevalence of antibodies against SARS‐CoV‐2 in the Norwegian population, August 2021

**DOI:** 10.1111/irv.13024

**Published:** 2022-06-30

**Authors:** Gro Tunheim, Gunnar Øyvind Isaksson Rø, Adity Chopra, Audun Aase, Anne‐Marte Bakken Kran, John Torgils Vaage, Fridtjof Lund‐Johansen, Olav Hungnes

**Affiliations:** ^1^ Division of Infection Control Norwegian Institute of Public Health (NIPH) Oslo Norway; ^2^ Department of Immunology Oslo University Hospital Oslo Norway; ^3^ Institute of Clinical Medicine University of Oslo Oslo Norway

**Keywords:** COVID‐19, infection, nucleocapsid, SARS‐CoV‐2, seroprevalence, vaccination

## Abstract

**Background:**

One year into the COVID‐19 pandemic, the cumulative number of confirmed COVID‐19 cases in Norway was still low. In January 2021, when the Norwegian COVID‐19 vaccination campaign started, the national seroprevalence estimate of SARS‐CoV‐2 antibodies was 3.2%. We have conducted a nationwide cross‐sectional study in August 2021 to investigate the overall prevalence of SARS‐CoV‐2 antibodies in Norway after 8 months of COVID‐19 mass vaccination and a third wave of SARS‐CoV‐2 infection.

**Methods:**

Residual sera were collected from laboratories across Norway in August 2021. In IgG antibodies against the spike protein, the spike receptor binding domain (RBD) and the nucleocapsid protein of SARS‐CoV‐2 were measured by a bead‐based flow cytometric assay.

**Results:**

In total, 1926 residual sera were collected from individuals aged 0–98 years; 55.1% were from women. The overall national estimated seroprevalence from vaccination and/or infection was 62.6% (credible interval [CrI] 60.1%–65.2%) based on having antibodies against both spike and RBD. Estimated seroprevalence increased with age. Among all samples, 11.7% had antibodies against nucleocapsid. For unvaccinated children <12 years, the seroprevalence estimate due to SARS‐CoV‐2 infection was 12.5% (95% CrI 9.3%–16.1%). Of seropositive samples from the unvaccinated children, 31.9% lacked anti‐nucleocapsid antibodies.

**Conclusions:**

The high overall SARS‐CoV‐2 seroprevalence estimates are in line with Norwegian registry data. Vaccination, not infection, contributed the most to the high seroprevalence in August 2021. Lack of antibodies against nucleocapsid should not automatically be interpreted as absence of previous infection as this could lead to underestimation of COVID‐19 cases in seroprevalence studies.

## 1 INTRODUCTION

Since the first case of coronavirus disease‐19 (COVID‐19) was reported in Norway in February 2020 and until August 2021, there had been three waves of COVID‐19 in Norway.^1^ However, due to strict non‐pharmaceutic interventions, the spread of SARS‐CoV‐2 infection in the Norwegian population had been quite limited.^2^, ^3^

The first COVID‐19 vaccine dose was administered in Norway on December 27, 2020, as part of a national mass vaccination campaign.^4^ Vaccination was prioritized to front‐line healthcare workers, elderly persons, and persons in risk groups, starting at the highest ages and with persons living in retirement homes.^5^ The vaccines used in the campaign were Comirnaty (BioNTech‐Pfizer, Mainz, Germany/New York, USA), Spikevax (mRNA‐1273, Moderna, Cambridge, USA), and Vaxzevria (AstraZeneca, Cambridge, UK). The latter was suspended in March 2021.^4^, ^6^

**FIGURE 1:**
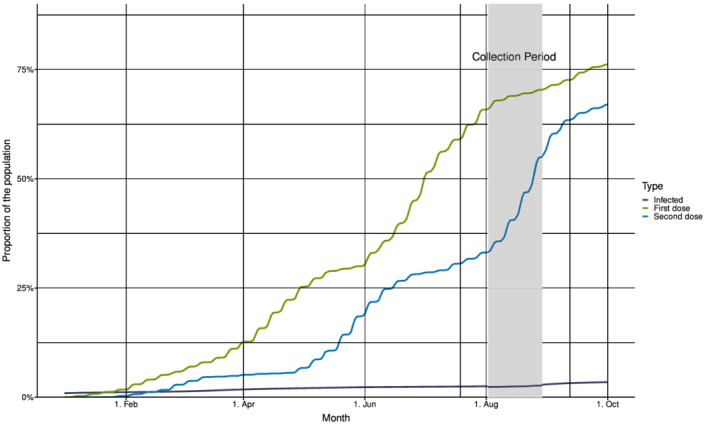
Cumulative incidence of confirmed COVID‐19‐cases and COVID‐19 vaccinations in Norway, January–October 2021, and timing of the residual sera collection. Incidence of cumulative infections (in dark blue) from Beredt C19 and frequency of COVID‐19 vaccinations (first dose in green and second dose in blue) from the Norwegian Immunisation Registry (SYSVAK). The collection period for sampling of residual sera (July 19–September 12) in the present study is indicated with vertical lines. The shaded area shows the period when 85% of the samples were collected.

By the end of July 2021, 135,993 cases of confirmed COVID‐19 had been reported to the Norwegian Surveillance System for Communicable Diseases (MSIS)^1^ (Figure 1). This corresponds to approximately 2.5% of the population. At the same time, 63% of the population (3,405,074 individuals) had been vaccinated with the first dose of COVID‐19 vaccine and 32% with the second dose according to the Norwegian Immunisation Registry (SYSVAK)^1^(Figure 1). For individuals aged ≥45 years and ≥65 years, 92% and 96% had received the first dose, respectively.

Prior to the start of the vaccine campaign, three nationwide cross‐sectional seroprevalence studies were conducted in Norway to monitor the development of the pandemic.^7^ In January 2021, at the start of the vaccine campaign, only 3.2% (95% credible interval [CrI] 2.3%–4.2%) of the Norwegian population had antibodies against SARS‐CoV‐2.^7^ We wanted to study the progression of SARS‐CoV‐2 seroprevalence after several months of the national COVID‐19 vaccination campaign and a third wave of SARS‐CoV‐2 infections. We also wanted to investigate whether we could distinguish between antibodies from vaccination or infection. Accordingly, we here present a nationwide cross‐sectional seroprevalence study based on residual sera collected in August 2021, that is, after approximately 8 months of COVID‐19 vaccinations. At the time of sampling, vaccinations of adults <45 years were still ongoing; most municipalities were vaccinating the population between 18–44 years. COVID‐19 vaccines were not approved for use in children under 12 years of age, and vaccination of 12–15‐year‐olds was not recommended until September 2021.

## 2 MATERIALS AND METHODS

### 2.1 Study samples

Anonymized residual sera were collected between July 19 and September 12, 2021, by 17 laboratories across Norway, as previously described.^7^, ^8^ Three samples were excluded due to antibody profiles indicating previous treatment with immunoglobulins. Sera lacking information on county of residence were attributed to the county of the submitting laboratory.

### 2.2 Antibody analysis

In IgG antibodies against the full‐length SARS‐CoV‐2 spike protein, the spike receptor binding domain (RBD), and nucleocapsid (N) protein were measured in a multiplex bead‐based flow cytometric assay as previously described.^9^, ^10^, ^11^Neutravidin‐coupled polymer beads with fluorescent barcodes and biotinylated virus proteins were incubated with serum. Subsequently, the beads were stained with R‐phycoerythrin‐conjugated anti‐human‐IgG‐Fc and analyzed by flow cytometry. The median fluorescence intensity (MFI) measured for each bead subset were divided by the MFI of beads with no antigen (blank). Cut‐off‐values were based on results from pre‐pandemic sera and samples from confirmed COVID‐19 convalescents with various disease severities. Seropositivity was defined as having antibodies against both spike and RBD (from vaccination and/or infection). In children aged <12 years (unvaccinated individuals), seroprevalence of infection was estimated based on either having antibodies against RBD and spike, or against RBD and nucleocapsid as in previous studies.^7^

A subset of the sera from children <12 years was also analyzed using spike S1‐protein SARS‐CoV‐2 IgG ELISA and SARS‐CoV‐2‐nucleocapsid IgG ELISA (Euroimmun, Lübeck, Germany). Borderline samples were considered positive (ratio optical density sample/internal calibrator ≥0.8).

### 2.3 COVID‐19 cases and COVID‐19 vaccinations

Aggregate data on COVID‐19 cases were obtained from the Emergency preparedness register for COVID‐19 (Beredt C19), which include data from the Norwegian Surveillance System for Communicable Diseases (MSIS). Aggregate data on COVID‐19 vaccinations were obtained from the Norwegian Immunisation Registry (SYSVAK). We used data on confirmed COVID‐19 cases and vaccinations from July 15, 2021, that is, approximately 3 weeks prior to the major sampling week. We included vaccinations and infections among the population with a valid national identity number and who were registered as residents in Norway in the National Population Registry.

### 2.4 Statistical methods

Seroprevalence was estimated for Norway and by age groups, sex, and county of residence following the same method as previously described.^7^ A Bayesian method that incorporates an uncertain sensitivity and an uncertain specificity of the antibody assay was used for the estimation.^12^ Based on validation data from known positive and negative cases, we estimated a sensitivity of 97% (CrI 95%–99%) and specificity of 99.7% (CrI 99.3%–99.9%) when defining seropositivity as having antibodies against RBD and Spike and a sensitivity of 95% (CrI 94%–98%) and specificity of 99.8% (CrI 99.5%–99.9%) when using antibodies against nucleocapsid and RBD. We corrected the overall seroprevalence by county, age group, and sex using a Bayesian multilevel regression post‐stratification (MRP) model.^12^ The seroprevalence estimates are presented as a point estimate and a 95% CrI. All Bayesian analyses were performed using Stan^13^ with the RStan interface.^14^ The fraction of positive samples as a function of age was estimated using a generalized additive model with a binomial link‐function and smoothing spline function with 20 knots for the age variable using the mgcv R‐package.^15^

### 2.5 Ethics

The residual sera were irreversibly anonymized at the laboratory of origin. Only aggregated data on confirmed COVID‐19 cases and COVID‐19 vaccinations were obtained from Beredt C19 and SYSVAK, respectively. The study was approved by the Regional Committee for Medical and Health Research Ethics in Southeastern Norway (reference number 157792).

## 3 RESULTS

A total number of 1926 residual sera were collected from individuals living in all 11 Norwegian counties by 17 different laboratories (102–120 samples per laboratory). Most samples (35.3%) were collected between August 2 and 8 and 84.6% of samples were collected between August 2 and 29, 2021 (Figure 1). Ninety‐five (4.9%) samples lacked information about the sampling week.

The residual sera were collected from individuals aged 0–98 years. The median age was 29 years, and 55.1% of the sera were from females. Of the sera 385 (20%) were collected from individuals younger than 12 years (Table 1). The age distribution of the study sera was slightly skewed towards younger ages compared with the Norwegian population (0–11: 14%; 12–17: 7%; 18–44: 36%; 45–64: 26%; ≥65: 18%).

**TABLE 1:** Estimated overall seroprevalence and corresponding percentages of COVID‐19 vaccinations and confirmed COVID‐19 cases by age, Norway

Age (years)	Positive samples	Number of samples tested (%)	Positive samples, % (95% CI)	Estimated seroprevalence, % (95% credible interval)	Percentage of the Norwegian population vaccinated with the first dose of COVID‐19 vaccine^a^	Percentage of confirmed COVID‐19 cases in the Norwegian population^b^
All	1029	1926 (100.0)	53.4 (51.2–55.7)	62.6 (60.1–65.2)^c^	58.4	2.3
0–11	47	385 (20.0)	12.2 (9.1–15.9)	12.5 (9.3–16.1)	0^d^	1.8
12–17	25	190 (9.9)	13.2 (8.7–18.8)	13.6 (9.0–18.9)	0.5	3
18–44	441	715 (37.1)	61.7 (58.0–65.3)	63.4 (59.6–67.1)	51.3	3.3
45–64	258	327 (17.0)	78.9 (74.1–83.2)	81.0 (76.0–85.6)	85.6	2.1
≥65	258	309 (16.0)	83.5 (78.9–87.5)	85.7 (81.2–90.0)	93.8	0.8

^a^ Data from SYSVAK, vaccinations by 15 July 2021.

^b^ Data from Beredt C19, confirmed COVID‐19 cases by 15 July 2021.

^c^ The overall seroprevalence for Norway was adjusted for age, sex and county.

^d^ COVID‐19 vaccines were not approved for use in children under 12 years.

Overall, 1029 (53.4%) of the residual serum samples were seropositive, giving an adjusted national seroprevalence from vaccination and/or infection of 62.6% (95% CrI 60.1%–65.2%; Table 1). Correspondingly, by July 15, 2021, approximately 3 weeks prior to the primary sampling week, 58.4% of the Norwegian population had received the first dose of COVID‐19 vaccine, and 2.3% confirmed COVID‐19 cases had been reported (Table 1).

The overall seroprevalence estimates for males and females were comparable (54.0% [95% CrI 50.3%–57.7%] and 55.4% [95% CrI 52.3%–58.6%], respectively). By July 15, 2021, 55.5% males and 61.4% females in Norway had been vaccinated with the first dose of COVID‐19 vaccine, while the percentages of confirmed COVID‐19 cases were 2.4% for males and 2.2% for females.

**FIGURE 2:**
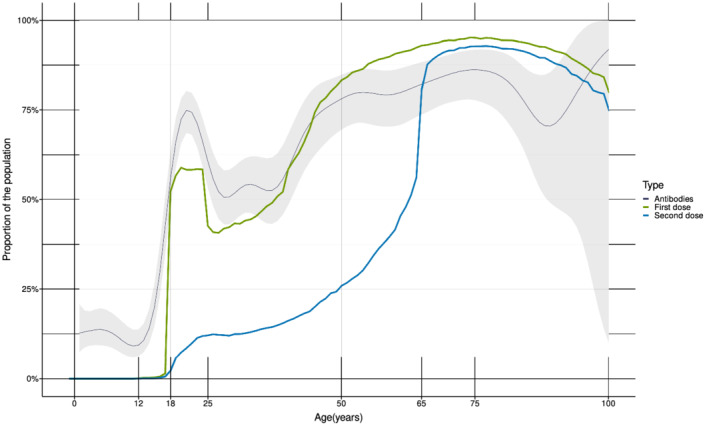
Percentage of seropositive study samples and percentage of COVID‐19 vaccinated individuals in Norway by age (years). The dark blue line shows the fraction of residual sera positive for antibodies against SARS‐CoV‐2 with the 95% confidence interval (grey area) (Seropositivity, not seroprevalence corrected for sensitivity and specificity). The green line shows the fraction of the Norwegian population vaccinated with the first dose of COVID‐19 vaccine and the blue line shows the fraction of the population vaccinated with the second dose of COVID‐19 vaccine by July 15, 2021 (data from SYSVAK). For individuals under 45 years, early vaccination was prioritized for the age groups 18–24 and 40–44 years over the age group 25–39 years.

The percentage of positive samples increased with increasing age (Table 1). The median age was 40 years (interquartile range [IQR] 23–63) for seropositive persons and 15 years (IQR 8–32) for the seronegative. The overall seroprevalence estimates were highest in the age groups ≥65 years (86%) and 45–64 years (81%), and low in the age groups below 18 years (<14%) (Table 1). The distribution of seropositive samples according to age corresponded well with the fraction of the Norwegian population vaccinated with the first dose of COVID‐19 vaccine by July 15, 2021 (Table 1 and Figure 2). However, for the age group 18–44 years, the estimated seroprevalence was higher than the percentage of vaccinated individuals, and the estimate was lower than the percentage of vaccinated individuals in the age group ≥65 years (Table 1). A rapid increase in seropositive study samples occurs around the age of 18 years, where the percentage of individuals vaccinated with the first dose also increases rapidly (Figure 2).

There were some small differences in the overall seroprevalence estimates from vaccination and/or infection between the 11 Norwegian counties (range 46%–63%) (Table 2). Children and teenagers were mostly unvaccinated at the time of sampling. Therefore, we also estimated seroprevalence for the different counties based on samples from individuals aged ≥18 years of age (Table 2). The seroprevalence estimates for the adult population were higher (range 60%–80%) and corresponded well with the percentage of individuals vaccinated with the first dose of COVID‐19 vaccine by July 15, 2021, for the various counties (Table 2).

**TABLE 2:** Estimated overall seroprevalence and corresponding percentages of COVID‐19 vaccinations and confirmed COVID‐19 cases by county of residence

County^a^	Positive samples	Number of samples tested (%)	Positive samples (all ages), % (95% CI)	Estimated seroprevalence (all ages), % (95% credible interval)	Positive samples (age ≥18 yrs), % (95% CI)	Estimated seroprevalence (age ≥18 yrs), % (95% credible interval)	Percentage of the population vaccinated with the first dose of COVID‐19 vaccine (age ≥18 yrs)^b^	Percentage of the population with confirmed COVID‐19 (age ≥18 yrs)^c^
Oslo	71	115 (6.0)	61.7 (52.2–70.6)	63.2 (54.2–71.9)	78.2 (68.0–86.3)	79.8 (70.0–88.5)	80.9	5.0
Rogaland	76	123 (6.4)	61.8 (52.6–70.4)	63.3 (54.3–72.0)	77.2 (66.4–85.9)	78.8 (69.0–87.7)	66.5	1.5
Møre og Romsdal	80	130 (6.7)	61.5 (52.6–69.9)	63.1 (54.1–71.8)	74.2 (68.5–79.3)	76.1 (70.4–81.5)	64.0	0.7
Nordland	62	126 (6.5)	49.2 (40.2–58.3)	50.6 (42.0–59.7)	61.7 (53.5–69.4)	63.2 (55.2–71.0)	63.3	0.6
Viken	222	390 (20.2)	56.9 (51.8–61.9)	58.4 (53.4–63.5)	67.0 (56.6–76.4)	68.5 (58.7–77.5)	80.8	3.4
Innlandet	66	121 (6.3)	54.5 (45.2–63.6)	56.1 (47.0–65.1)	70.9 (63.3–77.7)	72.7 (65.5–79.8)	65.9	1.5
Vestfold og Telemark	58	125 (6.5)	46.4 (37.4–55.5)	47.6 (38.8–56.5)	67.4 (56.7–77.0)	68.9 (58.6–78.5)	73.6	2.0
Agder	50	113 (5.9)	44.2 (34.9–53.9)	45.6 (36.4–55.0)	76.5 (66.9–84.5)	78.2 (68.8–86.6)	68.7	1.7
Vestland	124	246 (12.8)	50.4 (44.0–56.8)	51.8 (45.5–58.1)	73.9 (66.1–80.6)	75.7 (68.0–82.8)	70.2	1.5
Trøndelag	116	216 (11.2)	53.7 (46.8–60.5)	55.1 (47.9–61.9)	70.4 (59.2–80.0)	71.8 (61.3–81.6)	67.3	1.1
Troms og Finnmark	103	218 (113)	472 (405–541)	486 (417–554)	590 (473–700)	603 (494–711)	62.1	1.0
Missing	1	3 (0.2)	33.3 (0.8–90.6)	n.a^d^	50.0 (1.3–98.7)	n.a.	n.a.	n.a.

^a^ 8 sera were attributed to the county of the submitting laboratory as these sera did not have information on county of residence.

^b^ Data from SYSVAK, vaccinations by 15 July 2021.

^c^ Data from Beredt C19, confirmed COVID‐19 cases by 15 July 2021.

^d^ n.a.: not applicable. Seroprevalence was not estimated for subgroups with less than 30 samples.

### 3.1 Antibodies against SARS‐CoV‐2 due to infection

Among children 0–11 years (an unvaccinated population), 47 of 385 samples (12.2%) were seropositive, resulting in an estimated seroprevalence from SARS‐CoV‐2 infection of 12.5% (95% CrI 9.3%–16.1%) in this age group. The percentage of confirmed COVID‐19 cases in Norway was 1.8% for children <12 years and ranged between 0.8% and 3.3% for all age groups by July 15, 2021 (Table 1).

Samples positive for both RBD and spike were used to estimate overall seroprevalence, but we also considered other combinations of antibodies to see if they would allow a better estimate of the number of infections (Table 3). Spike‐based COVID‐19 vaccines do not induce antibodies against nucleocapsid. Overall, 226 samples (11.7%) had antibodies against nucleocapsid, which could suggest a previous SARS‐CoV‐2 infection (range 9%–14% for different age groups) (Table 3). We also found that 8.6% of all samples were positive for both nucleocapsid and RBD. In children <12 years, who had not received any COVID‐19 vaccines, we found that only 32 samples (8.3%) had antibodies against both nucleocapsid and RBD, while 47 samples (12.2%) had antibodies against both spike and RBD (Table 3). Consequently, in children <12 years, using antibodies against N and RBD as a definition of seropositivity resulted in a lower seroprevalence estimate of 8.7% (95% CrI 5.9%–11.8%) from COVID‐19 infection in this age group, compared with 12.5% (95% CrI 9.3%–16.1%) based on antibodies against both spike and RBD.

**TABLE 3:** Antibody responses to SARS‐CoV‐2 antigens by age groups. Positivity rates shown

Age (years)	Number of samples tested (%)	Positive samples, % (95% CI)		
		RBD^a^ and spike	Nucleocapsid	RBD and nucleocapsid
All	1926 (100.0)	53.4 (51.2–55.7)	11.7 (10.3–13.3)	8.6 (7.4–9.9)
0–11	385 (20.0)	12.2 (9.1–15.9)	11.4 (8.4–15.0)	8.3 (5.8–11.5)
12–17	190 (9.9)	13.2 (8.7–18.8)	14.2 (9.6–20.0)	6.3 (3.3–10.8)
18–44	715 (37.1)	61.7 (58.0–65.3)	13.3 (10.9–16.0)	9.1 (7.1–11.4)
45–64	327 (17.0)	78.9 (74.1–83.2)	9.5 (6.5–13.2)	8.6 (5.8–12.1)
≥65	309 (16.0)	83.5 (78.9–87.5)	9.4 (6.4–13.2)	9.1 (6.1–12.8)

^a^ RBD: Receptor binding domain of the spike protein.

Thus, despite not being vaccinated, in samples from 15 of the children below 12 years (31.9% of the seropositive samples in this age group), we found an antibody profile resembling the profile observed in vaccinated, uninfected individuals. These children had antibodies against spike and RBD, but not against nucleocapsid, and the results were confirmed for 12 of the 15 samples by spike S1 and nucleocapsid Euroimmun ELISAs (data not shown).

## 4 DISCUSSION

In this nationwide cross‐sectional seroprevalence study based on residual sera collected in Norway in August 2021, we report a high overall seroprevalence estimate from vaccination and/or infection of 62.6% (95% CrI 60.1%–65.2%). This finding corresponds well with the reported vaccination coverage of 58.4% for the first dose of COVID‐19 vaccine as well as the low percentage of confirmed COVID‐19 cases (2.3%) in Norway by July 15, 2021.

We observed differences in seroprevalence within the population, mostly according to age, but also according to geographical location. There was no difference in seroprevalence between males and females. The overall estimated seroprevalence was highest among the oldest age groups, which was expected, as vaccination was prioritized primarily based on age and given the overall low infection prevalence for all age groups in Norway.^4^ Among the residual sera from individuals ≥18 years, the percentage of seropositive samples closely resembled the fraction of the Norwegian population vaccinated with the first dose of COVID‐19 vaccine. However, for the age group 18–44 years, the seroprevalence estimate was higher than the percentage of vaccinated individuals in this age group. This is probably due to the highest incidence of confirmed COVID‐19 cases in this age group, as well as the rapid vaccination rate in this age group at the time when the sera were collected.^1^ For the older ages ≥65 year, the seroprevalence estimate was lower than the percentage of of individuals vaccinated in this age group. This observation may be due to immunosenescence or waning of vaccine‐induced antibodies,^16^ as the elderly were vaccinated early in 2021, at the start of the vaccination campaign.^17^

Vaccination started in Oslo in late December 2020, and within few weeks, vaccinations had commenced in all counties.^1^ Differences in vaccination coverage between counties can be explained by differences in the age distribution of the counties and that vaccine doses were prioritized to certain districts in Oslo and municipalities in Viken with a high incidence of COVID‐19 cases.^1^ However, the overall seroprevalence estimates of all counties were quite similar for individuals aged ≥18 years and resembled the reported percentage of individuals vaccinated with the first dose of COVID‐19 vaccines for most counties. The percentages of confirmed COVID‐19 cases varied but were generally low for all counties.

### 4.1 Distinguishing infection from vaccination

We report an estimated prevalence of SARS‐CoV‐2 antibodies (combination of spike and RBD) of 12.5% (95% CrI 9.3%–16.1%) in unvaccinated children under 12 years.^18^ This indicates an actual number of infections that was almost seven times higher than the number of confirmed COVID‐19 cases in this age group. However, the seroprevalence estimate due to SARS‐CoV‐2 infection in the unvaccinated children <12 years may not be directly extrapolated to older age groups because there may be differences in precautions to avoid getting infected and in symptoms and test activity. Moreover, vaccination of older age groups may have offered protection against infection, particularly in the first months after being vaccinated.^5^, ^19^

Antibodies against the nucleocapsid protein of SARS‐CoV‐2 have been used as an indication of previous infection with SARS‐CoV‐2, either alone or in addition to antibodies against spike/RBD.^7^, ^20^ Antibodies against nucleocapsid are also found after vaccine breakthrough infections. However, studies report a lower seroconversion rate against nucleocapsid if infection occurred after vaccination.^21^, ^22^, ^23^ We found that 11.7% of all the residual sera had antibodies against the nucleocapsid. This proportion is five times higher than the confirmed number of COVID‐19 cases in the general population, but similar to the seroprevalence estimate of infection in the unvaccinated children. However, cross‐reactive antibodies against the nucleocapsid of seasonal corona viruses^24^, ^25^ might lead to an overestimation of infection, whereas antibody waining^26^ or lack of seroconversion against nucleocapsid after breakthrough infection might lead to underestimation of infections. Among children <12 years who had not been vaccinated, we found a lower seroprevalence based on having antibodies against nucleocapsid and RBD compared to seroprevalence based on RBD and spike antibodies. This indicates that using a definition of seropositivity including the nucleocapsid likely would have underestimated the true seroprevalence in our sample. Antibody levels against SARS‐CoV‐2 antigens have been shown to wane over time since infection, particularly antibodies against nucleocapsid.^26^, ^27^, ^28^ Individuals with a poor antibody response to the nucleocapsid, might possibly seroconvert to undetectable levels sooner than individuals with higher levels.^27^ Consequently, based on measurements of nucleocapsid antibodies, samples from some individuals infected early in the pandemic could be categorized as negative (uninfected) in August 2021, 1.5 years into the pandemic. Indeed, the absence of antibodies against N‐protein in almost one‐third of the seropositive, unvaccinated children illustrates the limitations of using antibodies against nucleocapsid as a marker of previous infection. However, although the true cumulative percentage of SARS‐CoV‐2 infections in Norway probably was slightly higher than suggested by the measurements of antibodies against nucleocapsid in August 2021, it is still evident that the high overall seroprevalence estimate primarily originated from vaccination and not from SARS‐CoV‐2 infection.

Fifteen of the 47 seropositive samples from children <12 years had antibody profiles resembling the antibody profile of uninfected, vaccinated individuals. By the end of August 2021, only 30 children in Norway <12 years (0.004%) had received at least one dose of a COVID‐19 vaccine (personal communication with SYSVAK). Thus, it is very unlikely that residual sera from any of these children were included among the 385 study samples from children 0–11 years. Detection of antibodies against both spike and RBD therefore indicates a previous SARS‐CoV‐2 infection in these children, even in absence of antibodies against nucleocapsid. This antibody profile may have several explanations, related to differences in immune responses between children and adults, and to general features of antibody responses to nucleocapsid.

Some studies have found that children develop lower levels of antibodies against nucleocapsid than adults after SARS‐CoV‐2 infection.^29^, ^30^ Lumley et al. found that increasing age was associated with higher maximum anti‐nucleocapsid levels.^26^ In adults, antibody levels have also been shown to increase with increasing severity of infection,^28^, ^31^, ^32^, ^33^ although this has not been observed in children or adolescents.^30^, ^34^ Higher levels of IgG antibodies against spike or RBD compared to nucleocapsid have been observed in adult outpatients with mild disease.^35^ Lower levels of antibodies against nucleocapsid/lower rates of seroconversion against nucleocapsid have also been reported in COVID‐19 vaccinated individuals with breakthrough infections than after infection of unvaccinated individuals.^21^, ^23^, ^36^ Perhaps an earlier control of SARS‐CoV‐2 by the immune system in children or vaccinated individuals may result in a weaker antibody response against nucleocapsid.^21^, ^36^, ^37^, ^38^, ^39^, ^40^ The antibody assay can also influence detection of anti‐N antibodies.^33^ Here, lack of nucleocapsid antibodies in some of the seropositive samples from children were confirmed by a second method.

Based on our results and reports by others, we hypothesize that in some individuals, no or low levels of antibodies are induced against the nucleocapsid after SARS‐CoV‐2 infection. In combination with the documented faster waning of nucleocapsid antibodies, particularly in younger age groups,^23^, ^26^, ^29^ this implies that seroprevalence estimates of SARS‐CoV‐2 infection based on having anti‐nucleocapsid antibodies may underestimate the cumulative prevalence of SARS‐CoV‐2 infection. This may be the case specifically in children, but potentially also in highly vaccinated populations. Therefore, seroprevalence estimates of SARS‐CoV‐2 infection based on measurements of antibodies against nucleocapsid should be interpreted with caution. This has also been voiced as a concern by others.^21^, ^23^, ^27^, ^33^ Nevertheless, if these limitations are taken into consideration, measurements of anti‐nucleocapsid antibodies may contribute valuable information as a supplement to other measurements, as we report here.

The residual sera were collected anonymously. Consequently, it is not possible to link the seroprevalence results with the national registries of confirmed COVID‐19 cases or COVID‐19 vaccination. Moreover, the result cannot be linked with symptoms, severity of disease, timing of infection/vaccination, socioeconomic status, or behavioral factors. Norwegian COVID‐19 case numbers were increasing during the time of sampling and the COVID‐19 vaccination campaign was also still ongoing.^1^ Individuals recently infected or vaccinated with the first dose of COVID‐19 vaccine may not yet have developed antibodies at the time of sampling. Another limitation is that residual sera are collected from a population with potentially higher morbidity than the general population, as well as potential difference in health‐seeking behavior.^41^, ^42^ However, as an identical sampling strategy has been used several times during the pandemic,^7^ the estimates of these studies are comparable over time.

To conclude, we estimate a high overall seroprevalence of SARS‐CoV‐2 antibodies (62.4% [95% CrI 60.1%–65.2]%)) in Norway in August 2021. Both vaccination and infection contributed to the high seroprevalence; however, in agreement with Norwegian registry data, the national COVID‐19 vaccination campaign seems to have contributed the most. Further national seroprevalence studies may be useful for the continued surveillance of the pandemic and the impact of COVID‐19 vaccination. Lack of antibodies against nucleocapsid should not always be interpreted as absence of previous infection, and this assumption should be used with caution.

## CONFLICT OF INTEREST

None.

## ETHICS STATEMENT

The study was approved by The Regional Committee for Medical and Health Research Ethics in Southeastern Norway (Case number 157792).

## AUTHOR CONTRIBUTIONS

**Gro Tunheim:** Conceptualization; investigation; project administration. **Gunnar Rø:** Conceptualization; data curation; formal analysis; methodology; visualization. **Adity Chopra:** Investigation. **Audun Aase:** Conceptualization; investigation. **Anne‐Marte Kran:** Conceptualization; project administration. **John Torgils Vaage:** Methodology; resources. **Fridtjof Lund‐Johansen:** Data curation; formal analysis; methodology; resources; supervision. **Olav Hungnes:** Conceptualization; data curation; investigation; project administration; resources; supervision.

## Data Availability

The data that support the findings of this study are available from the corresponding author upon reasonable request.
